# Deploying FLAREs to Visualize Functional Outcomes of Host—Pathogen Encounters

**DOI:** 10.1371/journal.ppat.1004912

**Published:** 2015-07-09

**Authors:** Lena J. Heung, Anupam Jhingran, Tobias M. Hohl

**Affiliations:** 1 Infectious Diseases Service, Department of Medicine, Memorial Sloan Kettering Cancer Center, New York, New York, United States of America; 2 Immunology Program, Sloan Kettering Institute, Memorial Sloan Kettering Cancer Center, New York, New York, United States of America; McGill University, CANADA

## Functional Microbial Reporters in Host—Pathogen Studies

One of the most exciting features of fluorescent probes is their rapid evolution from simple tracers to functional indicators in diverse research fields [1]. In mammalian cells, fluorescence has been coupled to synaptic transmission [2,3], neuronal differentiation [4], apoptotic cell death [5], fate mapping in different immune cells [6,7], and functional changes in cell physiology, for example, the induction of cytokines [8]. In microbial cells, fluorescence has long been utilized as a tracer for pathogenic microbes, revealing microbial localization, residence, and spread in host tissues [9]. However, measuring functional outcomes during individual encounters with host immune cells remains challenging.

In this Pearl, we describe fluorescence-based approaches, which we term “functional microbial reporters,” designed to relay changes in microbial physiology that occur in host cell and tissue environments. This class of microbial reporters typically harnesses fluorescence emission at two wavelengths. One of these signals functions as an invariant tracer of microbial cells and is generally unaffected by hostile conditions that are encountered during interactions with host cells, such as reactive oxygen species or acidified compartments. The other signal varies according to a change in microbial physiology or residence in the host. This second signal may act as a ratiometric indicator (i.e., a shift in the excitation or emission spectrum) to reflect a continuous variable such as the microbial growth rate. Alternatively, the second signal may act as an on-off indicator (i.e., extinction in the emission spectrum) to reflect a binary variable such as microbial viability. Three specific types of functional microbial reporters will be discussed, including reporters that distinguish live and killed fungal cells [10], indicate the mode of host cell entry by an intracellular parasite [11], and measure bacterial growth rates in host tissues [12–14].

## Examples of Functional Microbial Reporters

**Fig 1:**
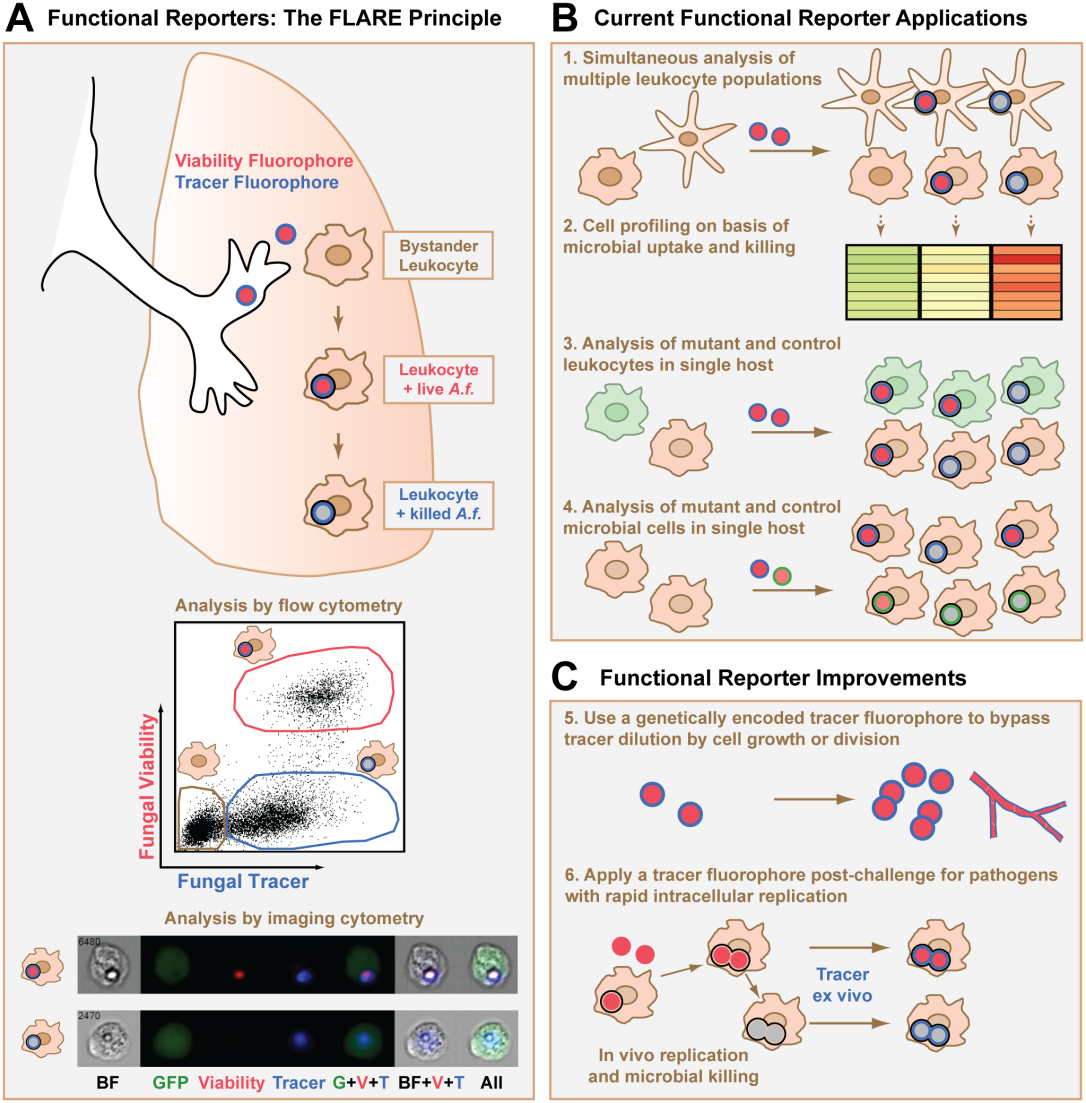
Design, applications, and potential improvements for a functional microbial reporter of viability. (A) Fluorescent *Aspergillus* reporter (FLARE) conidia represent an example of a functional microbial reporter. Live FLARE conidia emit two fluorescent signals: (i) a viability fluorophore DsRed (shown in red) that is extinguished at the time of conidial death, and (ii) a tracer fluorophore Alexa Fluor 633 (shown in blue) that persists after conidial death. A black ring indicates uptake of the conidium into the phagolysosome of a leukocyte. After experimental infection, flow cytometry can be used to distinguish bystander leukocytes (tan gate in flow cytometry plot) and fungus-engaged leukocytes that contain either live (red gate) or killed conidia (blue gate). Imaging cytometry can also be used to distinguish these different leukocyte groups. The imaging cytometry example shows green fluorescent protein (GFP)^+^ inflammatory monocytes that contain a live (top row) or killed (bottom row) FLARE conidium (image adapted from [20]). *A*.*f*., *Aspergillus fumigatus* conidium; BF, brightfield image. (B) Applications of a functional microbial reporter include (1) simultaneous analysis and (2) cell profiling of multiple leukocyte subsets on the basis of a microbial functional reporter readout, (3) simultaneous and focused analysis of microbial encounters with mutant and control leukocytes in a host with mixed chimerism, and (4) parallel analysis of the phenotype of mutant and control functional microbial reporters during cellular interactions with the immune system by using distinct tracer fluorophores for each microbial reporter. (C) Strategies to improve current functional microbial reporters include (5) creating functional microbial reporters in which the tracer and variant indicators are both genetically encoded so that the functional reporter is transmitted to progeny or (6) using microbial reporters that consist only of a variant indicator to infect the host and then staining with dyes or antibodies against the microbes for ex vivo quantification after the experimental conditions are complete.

The mold *Aspergillus fumigatus* is a major cause of infectious mortality in patients with acute leukemia and in hematopoietic and lung transplant recipients. Humans inhale many infectious conidia (non-dividing vegetative spores) daily, and a central function of the respiratory immune system is to prevent conidial germination into tissue-invasive hyphae. The molecular and cellular events that underlie conidial uptake and killing by leukocytes in the lung can be characterized using fluorescent *A* *spergillus* reporter (FLARE) conidia [10]. FLARE conidia are coupled to the dye Alexa Fluor 633 (AF633), which acts as an invariant signal independent of viability, and express DsRed as an on-off indicator of fungal viability (Fig 1A). Thus, live FLARE conidia emit AF633 and DsRed fluorescence and are readily visualized within leukocytes. FLARE conidia that have been taken up and killed by leukocytes only emit AF633 fluorescence since DsRed is extinguished in the phagolysosome [15], temporally coincident with conidial killing. In FLARE-infected mice, lung leukocytes can be analyzed on the basis of conidial uptake and viability using flow cytometry and imaging cytometry (Fig 1A), in addition to microscopy-based techniques.

*Toxoplasma gondii* is an obligate intracellular parasite that infects one-third of humans worldwide and causes disease primarily in immune compromised individuals. Parasites that actively invade host cells multiply within specialized parasitophorous vacuoles (PVs). However, parasites that have been engulfed by host phagocytes are contained within acidified phagolysosomes. Two functional microbial reporters have been used to distinguish active invasion from phagocytic uptake. In the first approach, non-replicating parasites express pH-insensitive mCherry and are loaded with Cell Trace Violet, a pH-sensitive dye that dims under low pH conditions [11]. Following murine challenge, host cells that contain parasites within PVs emit both fluorophores, while host cells that phagocytose parasites retain mCherry and lose Cell Trace Violet fluorescence. In the second approach, transgenic red fluorescent protein (RFP)^+^ parasites inject bacteriophage Cre recombinase into the host cell cytosol upon active invasion, but not upon phagocytic uptake [16]. Cre then induces GFP expression in cells from Cre-regulated GFP reporter mice, thus marking actively invaded host cells. These functional microbial reporters have been used to study differences in the induction of parasite-specific cytokines [17,18] and effector T cell responses [11] based on the mode of parasite entry.

*Salmonella* bacteria are a major cause of diarrheal illness and enteric fever. The emergence of antibiotic resistance by *Salmonella* species represents a public health threat. Two recently developed functional microbial reporters harness fluorescent protein dilution during cell division [14] or differences in the maturation rate of a DsRed variant called TIMER [12,19] to measure bacterial growth rates in antibiotic-treated mice. The latter reporter, termed TIMER^bac^-*Salmonella*, undergoes shifts in fluorescence over time from green to green/orange due to different kinetics of maturation and fluorescence resonance energy transfer between green and orange fluorophores [12]. These studies reveal that the majority of antibiotic survivors emerge from moderately growing, partially tolerant *Salmonella* [12], though non-replicating *Salmonella* contribute to the survivor pool as well [13,14].

## Current Applications of Functional Microbial Reporters

Functional microbial reporters enable a number of experimental readouts at the level of individual microbial cell—host cell encounters. First, different host leukocyte subsets can be analyzed in parallel during encounters with a functional microbial reporter (Fig 1B, point 1). This application identified lung-infiltrating inflammatory monocytes as effector cells that kill FLARE conidia directly during respiratory *A*. *fumigatus* challenge [20].

Second, host leukocytes can be profiled at the transcriptional or proteomic level based on a specific readout, such as microbial uptake and killing (Fig 1B, point 2), parasite mode of entry, or intracellular bacterial replication rate. Using this application, *T*. *gondii* entry into mononuclear phagocytes was found to be dispensable for protective interleukin-12 p40 release [18], while parasite phagocytosis, but not active invasion, induced protective type I interferon responses in mammalian hosts [17].

Third, functional microbial reporters provide a means to compare gene-deficient and control leukocytes side-by-side with regard to a defined microbial readout (Fig 1B, point 3). This application is particularly informative in mixed bone marrow chimeric hosts that contain gene-deficient and control hematopoietic cells that are distinguished by congenic markers. In this experimental setup, the impact of a genetic alteration in host cells on microbial functional outcomes is revealed in a leukocyte-intrinsic manner, independent of secondary effects on microbial burden and tissue inflammation that often arise in the analysis of globally gene-deficient mice.

Fourth, to compare wild-type and mutant microbial strains in a single host using a mixed infection model, it is possible to engineer functional microbial reporters with fluorescence signatures that differentiate between the two strains while using the same functional readout (Fig 1B, point 4). For example, mutant and wild-type fungal cells can be distinguished by incorporating a different tracer fluorophore, such as Brilliant Violet 421 [21], instead of AF633 in FLARE conidia while using the same viability fluorophore DsRed.

## Limitations and Possible Improvements of Functional Microbial Reporters

A key limitation of functional microbial reporters is the unwanted dilution of fluorophores that are not endogenously expressed. For instance, FLARE conidia have been an ideal model system for studying early innate antifungal immunity pathways that operate prior to the formation of fungal hyphae. However, the FLARE principle does not lend itself to the study of hyphae or other rapidly dividing microbes, since the exogenously applied tracer fluorophore is diluted as microbial cells germinate or divide. This limitation is apparent with the *Toxoplasma* mCherry-Cell Trace Violet reporter as well, since the pH-sensitive Cell Trace Violet dye is loaded into transgenic parasites that are replication-incompetent to avoid dilution of the pH indicator dye [11].

A potential solution to this limitation is to rely exclusively on fluorophores that are encoded in DNA, so that the fluorescent signals are self-replenishing as microbes divide or germinate (Fig 1C, point 5). This strategy was central to the generation of the *Salmonella* reporters of bacterial growth rate already discussed [12,14]. In the case of FLARE conidia, the use of a genetically encoded tracer fluorophore that does not quench after fungal death may improve the utility of these conidia and extend the FLARE principle to a broader range of pathogens, beyond spore-forming molds. For the *Toxoplasma* functional reporter, the use of genetically encoded pH-sensitive fluorescent proteins [2,22] anchored to the exterior parasite surface could conceivably replace the Cell Trace Violet dye and extend the functional reporter to replication-competent *Toxoplasma* strains. The feasibility of these genetic manipulations depends on the ease with which the target microbe can be mutated. Since genetically encoded fluorophores can be sensitive to environmental conditions, for example, ambient oxygen concentrations in host tissues [12], it is imperative to define the readouts of these functional microbial reporters under in vitro and in vivo conditions.

An alternative to genetically encoded tracer fluorophores is to use dyes or monoclonal antibodies to stain for all microbes, live or dead, in collected host tissues after the experimental conditions are complete (Fig 1C, point 6). For example, calcofluor white, a fluorescent dye for chitin found in fungal cell walls, could be used to reveal and quantify all fungal cells in permeabilized single cell suspensions from infected organs. Such an approach may extend the FLARE principle of coupling fluorescence to fungal viability to pathogenic fungi, like *Cryptococcus neoformans* or *Histoplasma capsulatum*, that grow as rapidly dividing yeast cells.

The kinetics of fluorophore degradation may limit the utility of functional reporters. In particular, if a viability indicator does not quench in a timely manner coincident with microbial death, significant outcomes may be overlooked. In experiments with recombinant *Escherichia coli*, DsRed degradation within *Dictiostylium discoideum* phagosomes occurred with a half-life of approximately 45 minutes [15]. Consistent with these results, the fluorescence signal in GFP-expressing *Plasmodium yoelii*-infected red blood cells was degraded with a half-life of 30–60 minutes in murine macrophage and dendritic cell phagosomes in vitro [23]. The mechanism by which DsRed and other genetically encoded fluorophores are quenched and degraded within mammalian phagosomes remains poorly defined. Therefore, the strict correlation between loss of fluorescence in sorted leukocyte populations and loss of microbial viability, as measured by colony-forming unit analysis, is critical to the success of the FLARE approach.

## Future Prospects of Functional Microbial Reporters

Although this Pearl highlights a small number of functional microbial reporters, the vast library of fluorescent protein indicators to monitor ion and metabolite concentrations, enzymatic activities, and the induction of microbial virulence factors suggests that this class of probes will expand rapidly at the host—microbe research interface. Coupling fluorescence to microbial properties that facilitate survival or immune evasion in host environments, such as dimorphism in specific fungi, antigenic hypervariability, or latency [24], will enable more precise understanding of the host—microbe dynamic in native tissue contexts. The examples presented herein collectively illustrate that functional microbial reporters facilitate molecular, cellular, and pharmacologic investigation into fundamental events that guide the outcomes of individual encounters between host and microbial constituents in vivo. Continued development of new and improved functional microbial reporters will be an important approach to improving our ability to decipher host responses to many clinically important pathogens.
